# Stretchable and High‐Performance Fibrous Sensors Based on Ionic Capacitive Sensing for Wearable Healthcare Monitoring

**DOI:** 10.1002/advs.202412859

**Published:** 2024-11-11

**Authors:** Jiawei Liu, Yan Yang, Guangchuan Chen, Hongbiao Sun, Xin Xie, Yanfeng Hou, Lishen Zhang, Jinhui Wang, Jiangxin Wang

**Affiliations:** ^1^ School of Mechanical Engineering Sichuan University Chengdu 610065 China

**Keywords:** electronic textiles, fiber, ionic liquids, iontronic sensors, stretchable electronics

## Abstract

Electronic textiles with remarkable breathability, lightweight, and comfort hold great potential in wearable technologies and smart human‐machine interfaces. Ionic capacitive sensors, leveraging the advantages of the electric double layer, offer higher sensitivity compared to traditional capacitive sensors. Current research on wearable ion‐capacitive sensors has focused mainly on two‐dimensional (2D) or three‐dimensional (3D) device architectures, which show substantial challenges for direct integration with textiles and compromise their wearing experience on conformability and permeability. One‐dimensional (1D) stretchable fiber materials serve as vital components in constructing electronic textiles, allowing for rich structural design, patterning, and device integration through mature textile techniques. Here, a stretchable functional fiber with robust mechanical and electrical performances is fabricated based on semi‐solid metal and ionic polymer, which provided a high stretchability and good electrical conductivity, enabling seamless integration with textiles. Consequently, high‐performance stretchable fiber sensors are developed through different device architecture designs, including pressure sensors with high sensitivity (7.21 kPa^−1^), fast response (60 ms/30 ms), and excellent stability, as well as strain sensors with high sensitivity (GF = 1.05), wide detection range (0–300% strain), and excellent sensing stability under dynamic deformations.

## Graphical abstract

A novel approach to develop stretchable iontronic sensors based on 1D stretchable fiber materials is presented, enabling their seamless integration with textiles through weaving or braiding process. Different device architectures are engineered into wearable textiles for wearable physiological signal monitoring and on‐body motion detections.

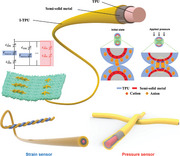

## 1 Introduction

Stretchable electronics, with high levels of breathability, comfort, and conformability, offer natural advantages in the fields of wearable devices for smart healthcare and interactive human‐machine interfaces.^[^ ^1^, ^2^, ^3^, ^4^, ^5^, ^6^ ^]^ Among these, electronic textiles, characterized by their high robustness, lightweight nature, breathability, washability, and inherent compatibility with the mature textile technologies, have garnered widespread attention in wearable sensors,^[^ ^7^, ^8^, ^9^, ^10^ ^]^ energy harvesters,^[^ ^11^, ^12^, ^13^, ^14^ ^]^ displays,^[^ ^15^, ^16^ ^]^ and actuators.^[^ ^17^ ^]^ As crucial and fundamental building blocks of electronic textiles, 1D stretchable fiber materials enable seamless integration and patterning through textile processes and possess significant potential in wearable sensing applications due to their large specific surface area and high mechanical compliance. Despite the notable progress in the fabrication techniques such as layer‐by‐layer deposition,^[^ ^18^ ^]^ wet spinning,^[^ ^19^ ^]^ electrospinning,^[^ ^20^ ^]^ and thermal drawing,^[^ ^21^, ^22^ ^]^ etc. for 1D material preparation, the development of stretchable functional fiber materials and their integrations with textiles still require extensive research endeavors to enable high‐performance wearable electronics.

Capacitive sensors, featuring with high sensitivity, high linearity, and rapid response, are highly suitable for the development of electromechanical sensors used in applications such as on‐body healthcare monitoring^[^ ^23^, ^24^ ^]^ and conformable human‐machine interactions.^[^ ^22^, ^25^ ^]^ Conventional capacitive sensing systems used for strain^[^ ^14^, ^23^, ^26^, ^27^ ^]^ and stress^[^ ^22^, ^25^, ^26^, ^28^, ^29^, ^30^ ^]^ detection have demonstrated limited sensing performances. In comparison, Ion‐capacitive sensors based on the electric double layer (EDL) have shown promising performances with enhanced sensitivity and sensing range.^[^ ^11^, ^31^, ^32^, ^33^, ^34^ ^]^ Moreover, the EDL contributes a large capacity in the devices, with capacitance values typically three orders of magnitude higher than those of traditional dielectric materials‐based capacitors, providing higher immunity to interference and offering new avenues for self‐powered sensor development.^[^ ^35^ ^]^ Wu et al. fabricated an all‐fiber ionic stretchable pressure sensor exhibiting high sensitivity (1.08 kPa^−1^) across a broad range (0‐300 kPa) and rapid response and recovery times (≈18/22 ms) through electrostatic spinning and stencil printing techniques.^[^ ^34^ ^]^ Lin et al. developed an ionic capacitance sensor with a three‐layer nanofiber membrane structure, which exhibits high sensitivity (217.5 kPa^−1^) in the pressure range of 0–5 kPa.^[^ ^11^ ^]^ Although existing sensors based on electric double layer capacitor (EDLC) have shown high sensing performances, most research has been limited on 2D or 3D device architectures such as electronic skin patches and infiltrated fabrics. These devices make it difficult to maintain the high conformability and moisture permeability of textiles for long‐term and comfortable wearable applications. Research on 1D fiber materials for ion capacitance sensors, particularly those with robust performances under large mechanical deformations, warrants further exploration and development.

In this work, we propose a strategy to fabricate stretchable fibers for ionic capacitive sensing, which enabled seamless integration with textiles for highly conformable on‐body electronic applications. The fiber consists of a central support layer of elastic material, an intermediate electronic layer of semi‐solid metal with high stretchability (>700%) and good electrical conductivity (≈4537.28 S cm^−1^), and an outer iontronic layer of ionic polymer. With weaving or braiding approaches, the stretchable fibers were integrated with textiles to demonstrate elastic pressure or strain sensors. The pressure sensors designed based on the fibers exhibit high sensitivity (7.21 kPa^−1^), fast response and recovery times (60 ms/30 ms), low response thresholds (≈28.32 Pa), and stable performance under repeated stretching (≈100%) conditions. The strain sensor exhibits high sensitivity (GF = 1.05), a wide sensing range (0–300% strain), excellent cyclic stability, and good response to dynamic deformations. Taking advantage of the excellent performance of stretchable ionic capacitive fiber sensors, we were able to demonstrate various electronic textile applications, including wearable physiological signal monitoring such as heartbeat, respiration, and swallowing with the fibrous pressure sensors and on‐body motion detections for finger, wrist, and knee monitoring with the fibrous strain sensors.

## 2 Result and Discussion

### 2.1 Performances of the SICF

**Figure 1:**
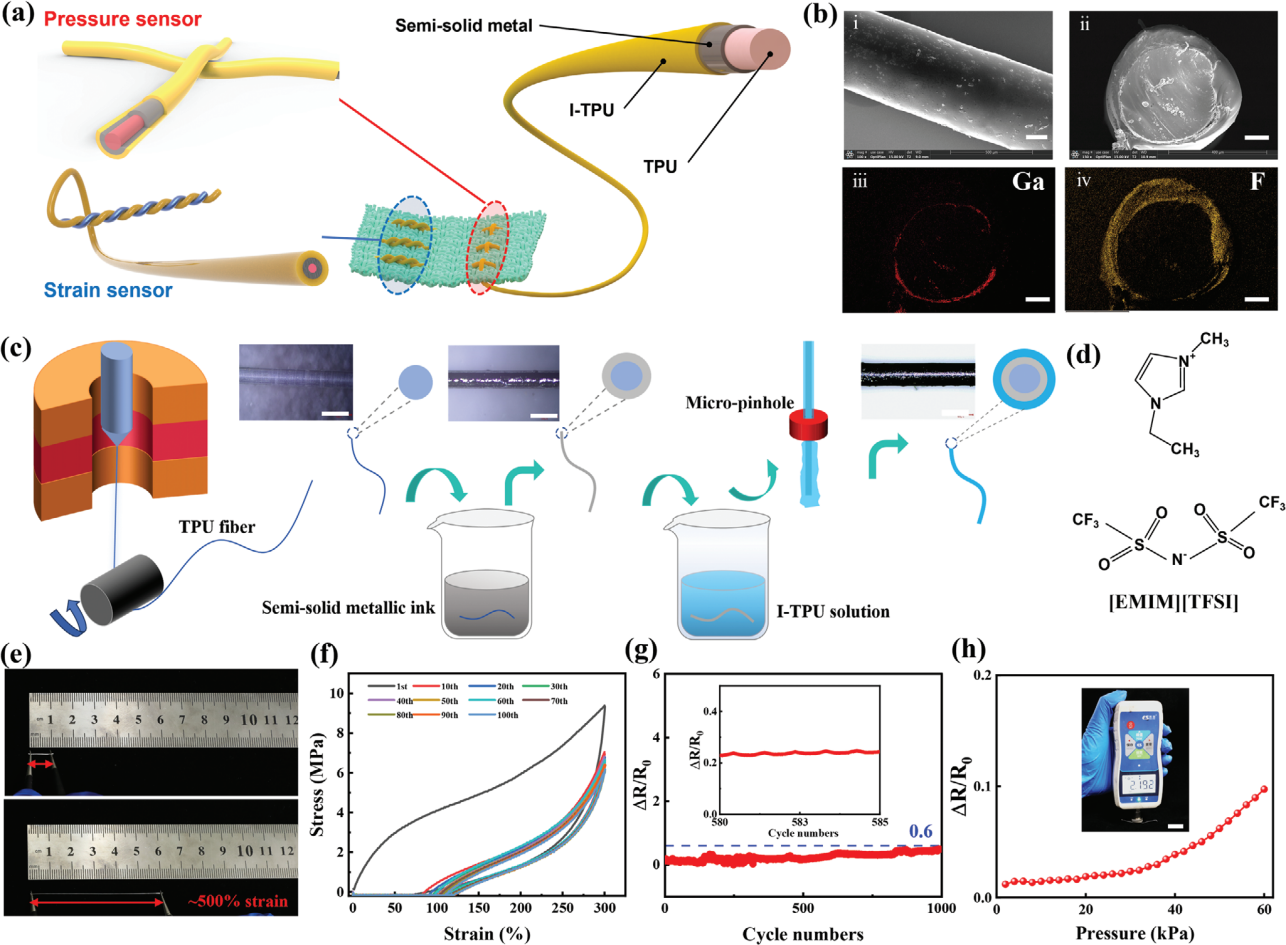
3D structure, manufacturing process, and electromechanical properties of fibers: a) Schematic illustration of the sensing textile based on SICFs with a triple‐layer structure. And schematic illustration of the device architectures for the pressure sensor and the strain sensor. b) SEM images of (i) the surface and (ii) the cross‐sectional microcosmography of SICF. (iii‐iv) EDS elemental mapping of SICF. Scale bar, 0.1 mm. c) Schematic of the preparation process for SICFs. Scale bar, 0.5 mm. d) Chemical formula of the ionic liquid. e) Optical photographs of the fiber in its original state (top) and stretched state with ≈500% strains (bottom). f) Cyclic loading‐unloading curves at a fixed tensile strain of 300% for 100 cycles. g) The resistance variations of a SICF sample under a tensile strain of 100% for 1000 cycles. h) The resistance variations of a SICF sample under compressive strains. Scale bar, 2 cm.

The 3D structure presented in **Figure** **1a** illustrates the triple‐layer structure of the stretchable ionic capacitive fiber (SICF), along with schematic representations showing the fibers woven into the textile. An interlaced structure was designed for the pressure sensor and a helical structure was designed for the strain sensor, respectively. The scanning electron microscopy (SEM) images depicted in Figure 1b(i,ii) show the surface and cross‐sectional characteristics of the SICF. The energy dispersive spectroscopy (EDS) images depicted in Figure 1b(iii,iv), alongside with Figure S1 (Supporting Information) distinctly elucidates the trilaminar configuration of the fibers. The fibers were obtained through a layer‐by‐layer coating process. Figure 1c shows the illustrations of the layer‐by‐layer process for preparing SICFs, and the insets show the optical micrographs of the TPU fiber, fiber coated with semi‐solid conductive inks, and SICF respectively. Thermoplastic Polyurethane (TPU) was chosen as an elastic material for SICF due to its exceptional electromechanical properties and ease of processing. The TPU fibers are obtained through the thermal drawing process, serving as the core fiber for the SICF. Diameters of the TPU fibers can be tailored by controlling the heating temperature and drawing speed for different requirements.^[^ ^36^ ^]^ During the thermal drawing process, the preform is fed into the furnace at a constant feed speed (ν_feed_), while the fiber is drawn by a rotating winder at different drawing speed (ν_draw_). Following the conservation of volume, when the feed speed remains constant, the fiber diameter (*D*_fiber_) decreases as the drawing speed increases, where the diameter of the preform is represented by *D*_preform_.^[^ ^36^ ^]^

(1) $$D_{\mathrm{fiber}}=D_{\mathrm{preform}}\;\;\sqrt{\frac{\nu_{\mathrm{feed}}}{\nu_{\mathrm{draw}}}}$$

Diameter of the TPU fibers used to assemble the SICFs was 0.4 mm in this work. The conductive electrode, situated in the intermediate layer, comprised a semi‐solid metallic ink, which was prepared by homogenizing the mixture of liquid metal (LM) and nickel (Ni) particles. The incorporation of metal particles has been demonstrated to be an effective strategy for modulating the surface tension and viscosity of liquid metals.^[^ ^37^, ^38^ ^]^ The incorporation of nickel particles resulted in a notable reduction in the contact angle from 110° to 50° (Figure S2, Supporting Information) and a significant increase in the adhesion (Figure S3, Supporting Information) of the liquid metals on the TPU film substrate. As illustrated in Figure S3c (Supporting Information), compared to the pure LMs, the Ni‐LMs can be uniformly and continuously coated onto the TPU fibers due to their low surface tension and excellent adhesion. The solid‐state electrolyte, acting as the iontronic layer for the capacitive sensors and the protection layer for the semi‐solid metallic electrode, was comprised of ionic TPU (I‐TPU), which was prepared by mixing ionic liquid [EMIM][TFSI] (Figure 1d) and TPU. During the coating process, control over the overall dimensions of the fibers was achieved through the utilization of a miniature pinhole.

Figure 1e presents photographs of the SICF in its original state and under ≈500% tensile strain, showing its good stretchability. Figure S4 (Supporting Information) illustrates the stress‐strain curve of the SICF under stretching, which shows the excellent tensile properties of SICF with an ultimate stretching strain of up to 760%. The good mechanical recovery of SICF was demonstrated by the tensile test with a fixed strain of 300% for 100 cycles, which exhibited nearly overlapping stress‐strain curves after the first cycle where the irreversible stress relaxation occurred, as shown in Figure 1f. Furthermore, we investigated the electrical performances of the semi‐solid electrode in the SICF under tensile and compressive strains, considering the stable conductivity of the electrode is of great importance for the SICF sensing performance. Figure 1g shows the resistance changes of the SICF under a tensile strain of 100% for 1000 cycles. It can be observed that the ΔR/R_0_ of the semi‐solid electrode is less than 0.01 within a single stretching cycle and maintains below 0.6 after 1000 cycles of stretching. Figure 1h demonstrates that the ΔR/R_0_ of the semi‐solid electrode remains below 0.1 within the applied pressure range of 0 to 60 kPa. The excellent resistance stability of the electrode in SICF provides crucial assurance for its application to enable high‐performance electromechanical sensors, which provide stable sensing behaviors under large mechanical deformations.

### 2.2 Pressure Sensors Based on SICF

**Figure 2:**
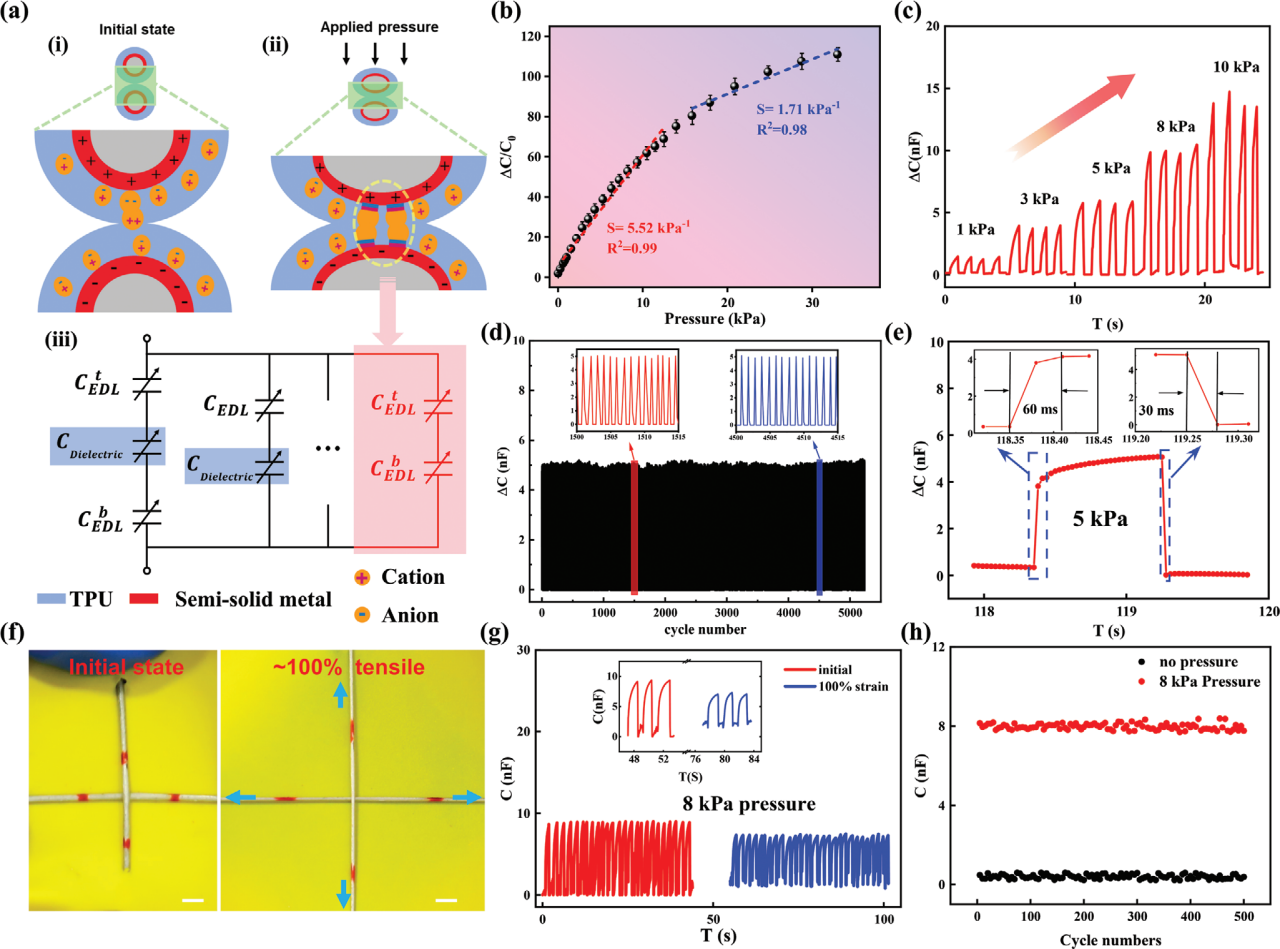
Working mechanism and performance of SICF‐based pressure sensors: a) The model diagrams in the initial (i) and pressure (ii) state and the equivalent circuit diagram (iii) of the ionic capacitor. b) Change in capacitance of a pressure sensor as a function of applied force. c) The response curve of the sensor under different pressures. d) Mechanical durability test of the sensors for more than 5000 loading/unloading cycles at a fixed pressure of 5 kPa. e) Response and recovery time under a pressure of 5 kPa. f) Photographs of the sensor at the initial state (left) and stretching state with 100% tensile strain (right). Scale bar, 2 mm. g) The capacitance response of the sensors to 8 kPa pressure in the initial (red) and 100% stretched (blue) states. h) The capacitance of the sensor to 8 kPa pressure at the end of each cycle throughout 500 cycles of 100% strain stretching.

As shown in Figure 1a, the stretchable pressure sensor comprises a basic unit consisting of an interlaced structure. Under stress, the cross nodes experience increased compression, which leads to changes in the contact area of the fibers, resulting in variations in capacitance. The remarkable capacitive properties of ionic capacitive sensing can be attributed to the EDL, which typically occurs at the solid‐liquid and solid‐solid electrification interface.^[^ ^39^, ^40^ ^]^ In this study, electrons within the semi‐solid metal layer and ions of opposite charge within the I‐TPU layer attract each other and form a double layer at the interface. **Figure** **2a** shows the schematic and an equivalent circuit of the ionic capacitor. In the initial state, as illustrated in Figure 2a(i), the two fibers exhibit loose contact with a small contact area and a large inter‐electrode distance. In this configuration, as the ions cannot fill the entire polymer network, EDLs between the two electrodes were only created in localized regions. The system can be considered as an capacitance (C_0_) with EDL capacitances and the dielectric capacitances connected in series, as delineated by:

(2) $$C_{0}=\sum \frac{1}{\;\frac{1}{C_{EDL}^{t}}+\frac{1}{C_{Dielectric}}+\frac{1}{C_{EDL}^{b}}\;}\approx \sum C_{Dielectric}$$

Specifically, $C_{EDL}^{t}$ and $C_{EDL}^{b}$ are the EDL capacitances formed at the positive and negative electrode interfaces, respectively, while *C_Dielectric_* is the dielectric capacitance of the TPU network. Given that *C_Dielectric_* is markedly smaller than *C_EDL_* due to the minimal spacing between charges within the EDL capacitor^[^ ^13^, ^40^ ^]^ and considering their series connection, the overall capacitance is predominantly determined by *C_Dielectric_*, yielding a low initial capacitance.

Upon the application of pressure, as shown in Figure 2a(ii), the fibers come into closer contact with increased contact areas and reduced distances between the electrodes. The pressure‐induced ion penetration into the polymer network established continuous ionic pathways within the contact region.^[^ ^30^ ^]^ The total capacitance (*C*_1_) is dominated by the EDL capacitance, as formulated in Equation 3, experiencing a significant increase. Additionally, a further increase in pressure will expand the EDL region and further elevate the capacitance.

(3) $$\begin{array}{ccc} C_{1} & = & \sum \frac{1}{\;\frac{1}{C_{EDL}^{t}}+\frac{1}{C_{EDL}^{b}}\;}+\sum \frac{1}{\;\frac{1}{C_{EDL}^{t}}+\frac{1}{C_{Dielectric}}+\frac{1}{C_{EDL}^{b}}\;}\\ & & \approx \sum C_{EDL}+\sum C_{Dielectric}\approx \sum C_{EDL} \end{array}$$

**Figure 3:**
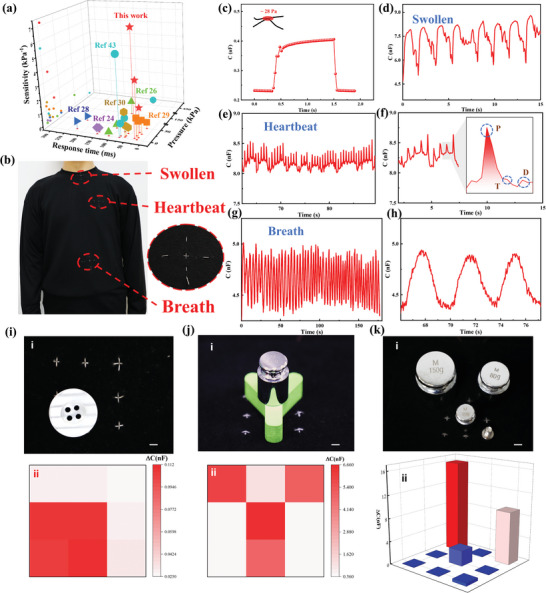
The application of SICF‐based pressure sensors in physiological signal monitoring and sensing arrays: a) Comparing the response time and sensitivity under different pressures with capacitive sensors from previous reports. b) The picture of sensor arrangement on clothing. The magnified picture of the sensor sewn into clothing. Scale bar, 1 cm. c) Capacitance change at a pressure of ≈28 Pa. d) The capacitance curve of the stretchable sensor based on SICF during swallowing. e) The capacitance curve of the stretchable sensor used for monitoring a heartbeat and f) its enlarged portion. g) The capacitance curve of the stretchable sensor used for monitoring respiration and h) its enlarged portion. i) Photographs (i) and capacitance response curves (ii) of a 3*3 pressure sensing array for a clothing button sensing. Scale bar, 3 mm j) Photographs (i) and capacitance response curves (ii) of the pressure sensing array for Y‐shaped block sensing with an additional load of 30g. Scale bar, 3 mm. k) Photographs (i) and capacitance response curves (ii) of the sensing pressure array for 5 g, 20 g, 80 g, and 150 g weight sensing. Scale bar, 3 mm.

Figure 2b presents the capacitance response of the pressure sensor, composed of 0.5 mm diameter SICFs, within the 30 kPa pressure range. The sensitivity of the pressure sensor can be defined as the slope of the traces in Figure 2b, which exhibits three distinct regions with varying values. It is observed that the sensitivity decreases with increasing pressure. The linearity is above 0.98 in the three regions. For pressures below 6 kPa, between 6 and 166 kPa, and between 16 and 30 kPa, the sensitivities are 7.21 kPa^−1^, 3.65 kPa^−1^, and 1.54 kPa^−1^, respectively. The diameter of the fibers plays a crucial role in determining sensor sensitivity (Figure S5, Supporting Information). Figure 2c illustrates the response curve of the sensor under different magnitudes of pressure. It can be observed that the sensor demonstrates stable responses to the same pressure, and the capacitance increases with the incremental force, ranging from 1 to 10 kPa. The response curve of the sensor, subjected to a fixed pressure of 5 kPa for over 5000 cycles, is shown in Figure 2d, demonstrating the excellent stability and durability of the sensor. Furthermore, the sensor exhibits a swift response to lower pressures, with response and recovery times to a 5 kPa pressure of 60 ms and 30 ms respectively, as shown in Figure 2e. Compared to similar works,^[^ ^28^, ^29^, ^30^, ^41^, ^42^, ^43^ ^]^ the SICF‐based pressure sensor demonstrates significant advantages in sensing sensitivity and response speed, as shown in **Figure** **3a** and Table S1 (Supporting Information). To further investigate the response of the pressure sensor under tensile deformation, it was subjected to a 100% bidirectional strain, as shown in Figure 2f. The sensing response was measured before and after the cyclic elongation. As shown in Figure 2g, under the 100% bidirectional strain, the fiber remains excellent response to the applied pressure. However, there is a slight increase in initial capacitance compared to the unstretched state, which is attributed to that additional pressure was applied onto the sensor due to stretching. Additionally, in the stretched state, the sensor's capacitance response slightly decreases in response to the same magnitude of force. This is due that the elongation caused a reduction in fiber diameter, which in turn reduces the area of the EDL and consequently decreases the capacitance response. Although the sensor's response exhibits slight variations under a stretched state, the variations might be addressed through designed algorithms, ensuring accurate readings.^[^ ^44^ ^]^ In the realm of wearable device applications, the devices are typically subjected to cycles of stretching and relaxation. As illustrated in Figure 2h, the sensor response to a force of 8 kPa was tested after each cycle throughout the cyclic stretching test (500 cycles of 100% strain). Impressively, the sensor maintains excellent stability and sensing accuracy after repeated stretching, demonstrating its robust mechanical durability.

Monitoring of human physiological signals plays a crucial role in clinical diagnosis and rehabilitation efforts. Particularly, long‐term monitoring can provide more accurate diagnostic data for chronic diseases, which poses a significant challenge to conventional wearable devices. Fiber‐based sensors, which can be directly woven into wearable textiles, offer excellent moisture permeability and high conformability for intimate contact, facilitating long‐term and continuous biosignal monitoring. Based on the excellent performance of the pressure sensor, particularly its high sensitivity to small pressures (Figure 3b), it can be well integrated into clothing for multiple physiological signal monitoring purposes, as illustrated in Figure 3c. The digital photograph in Figure 3c provides further insight into the details of the sewing structure for the device integration.

Neuromuscular disorders such as stroke and Parkinson's disease can lead to abnormal muscle spasms and impair swallowing function. Monitoring of swallowing behavior can effectively assist patients in postoperative rehabilitation training. Figure 3d shows the response of a stretchable swallow sensor based on SICF, with capacitance changes induced by the movement of muscles in the throat during swallowing. Continuous monitoring of human heart rate is a critical method for diagnosing and preventing cardiovascular diseases. Figure 3e demonstrates the dynamic sensing curve of the pressure sensor positioned on the left chest of the user, monitoring a heartbeat over 200 s. From the enlarged curve in Figure 3f, it is evident that the sensor is capable of monitoring the three primary waveforms of cardiac pulsation: P(percussion)‐wave, T(tidal)‐wave, and D(diastolic)‐wave. Analysis of these characteristic waveforms' variations can provide diagnostic insights into the cardiac health status. Respiration sensing is also crucial in clinical monitoring, particularly for patients with severe medical conditions. Nighttime respiratory signals serve as an effective evaluation reference. Figure 3g presents the respiratory signals detected using the SICF‐based sensors over 160 s. Figure 3h shows an enlarged cure of the captured signal. The variations in capacitance primarily result from the abdominal movements during the respiratory process. During inhalation, the abdomen rises and compresses the sensor, leading to an increase in capacitance values, whereas, during exhalation, relaxation causes a decrease in capacitance values.

To further demonstrate the versatility of fibrous sensors to enable different device architectures in electronic textiles, we constructed a 3×3 array sensor with 9 pixels by weaving fibers into cotton fabric to detect the spatial distribution of the load pressure. Figure 3i presents the photograph (i) and response (ii) of the pressure sensor array to a button weighing 2.5 g. The color differences illustrate the magnitude of capacitance changes each pixel experiences, indicating that the pressure sensor array can identify the spatial distribution of even minute forces. Figure S6 (Supporting Information) and Figure 3j show the pressure response before and after adding a 30 g weight to a Y‐shaped module weighing 3.5 g, respectively. By comparing the results before and after adding the load, it can be observed that the pressure sensor array not only identifies the spatial distribution of pressure but also maintains a high resolution in determining the force magnitude. Additionally, we tested its response to 5 g, 20 g, 80 g, and 150 g weights, as illustrated in Figure 3k, demonstrating the pressure sensor array's stability and sensitivity within a broad pressure range.

### 2.3 Strain Sensors Based on SICF

**Figure 4:**
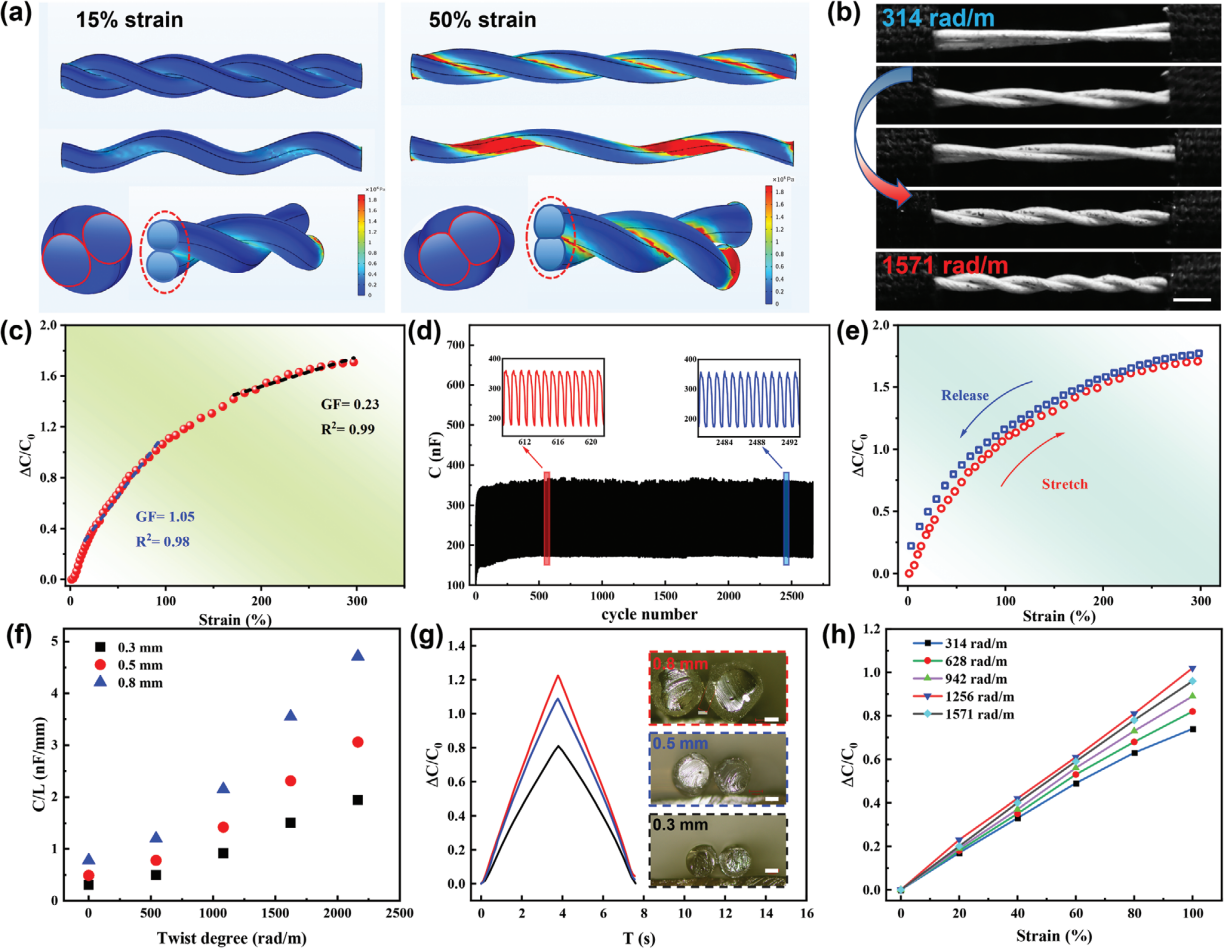
Structure and performance of SICF‐based strain sensors: a) Numerical simulation of stress distribution in helical fibers at 15% strain (left) and 50% strain (right). b) Photographs of fibers at different twist degrees. c) Change in capacitance of the sensor as a function of strain. d) Capacitive response under cyclic loading and unloading at 100% strain for 2500 cycles. e) Change in capacitance of the strain sensor after the 2500 stretching cycles with a stretch‐release test at 300% strain. f) Capacitance of sensors composed of different twist degrees and fiber diameters. g) Relative changes in capacitance of strain sensors with different fiber diameters under 100% strain. h) Relative changes in capacitance of strain sensors with different twists under 0–100% strain range.

Interestingly, the SICF can also be configured into a spiral‐twisted structure to achieve a strain sensor. **Figure** **4a** illustrates the deformation and stress simulation results of the helical fibers under 15% and 50% tensile strains. The portion of the two fibers in contact is equivalent to a twisted rectangular surface, with the length of which increasing with the axial stretching of the fibers. Furthermore, as the strain increases, the two fibers come into closer contact, which leads to an increase in the width of the rectangle and hence a further increase in the contact area. Figure S7 (Supporting Information) illustrates the photographs of helical fibers under 50% and 100% tensile deformation. Based on the double‐layer effect described in Figure 2a, the increase in contact area leads to an increase in capacitance. The capacitance change curve with strains in Figure 4c demonstrates that the strain sensor achieved a gauge factor (GF) of 1.05 with a linearity of 0.98 within 100% strain range, highlighting the excellent sensitivity and linearity of the helical fiber strain sensor. Furthermore, the sensor's maximum strain sensing limit was up to 300%, while maintaining a GF of 0.23 with a linearity of 0.99 at high strain levels. The gauge factor (GF) is a parameter that quantifies the sensitivity of a strain sensor and is calculated using the formula:

(4) $$GF=\frac{\Delta C}{C_{0}\cdot \;\varepsilon }$$

Where *ε* is the strain applied on the fiber, *C_0_* is the original capacitance, and *ΔC* is the capacitance change under strain.

Moreover, the long‐term stability of the sensor was evaluated over 2500 cyclic stretching tests at 100% strain, as illustrated in Figure 4d. The results demonstrated that the sensor exhibits excellent cyclic stability and durability. To further verify the repeatability and stability of the fiber strain sensor, we examined its performance after the 2500 stretching cycles with a stretch‐release test at 300% strain, as shown in Figure 4e. The delay hysteresis (DH) value, a key parameter for quantifying hysteresis, can be calculated using Equation 1, where *A*_loading_ and *A*_unloading_ are the areas under the sensor's capacitance change versus strain curve during loading and unloading, respectively:^[^ ^45^ ^]^

(5) $$DH=\frac{A_{\mathrm{loading}}-A_{\mathrm{unloading}}}{A_{\mathrm{loading}}}\times 100\%$$

The sensor exhibited minor hysteresis with a DH of ‐3.95%, confirming its stable performance over mechanical deformations. Although the sensitivity of the SICF capacitive sensor is lower compared to piezoresistive strain sensors,^[^ ^10^ ^]^ it exhibits superior sensing linearity and reversibility.

Since the strain sensors were fabricated by helically twisting two fibers, the fiber diameter and the degree of twisting are expected to have a significant influence on the sensor's performance. As shown in Figure 4f, we evaluated the capacitance performance of fibers with diameters of 0.3 mm, 0.5 mm, and 0.8 mm under different degrees of torsion. The initial capacitance of the fiber increases with the degree of twist. This is primarily because the higher twist ratio reduces the distance between the fibers, resulting in a larger effective contact area that increases the capacitance. Besides, sensors composed of fibers with larger diameters demonstrate higher sensitivity, as shown in Figure 4g. This is because larger fibers contribute greater modifications at the fiber‐to‐fiber contact areas when stretched, leading to higher changes in capacitance. As illustrated in Figure 4b, sensors with twisting ranging from 314 to 1571 rad m^−1^ were examined under 0–100% strain to explore the effect of twisting on sensing sensitivity. The results in Figure 4h show that sensitivity and linearity of the sensor increases with the degree of twist up to a certain point. Specifically, sensors with a twist ratio of 1256 rad m^−1^ exhibit optimal sensitivity. However, when the degree of twist exceeds this threshold, the sensitivity begins to decrease. This phenomenon could be attributed to that excessive twisting increases the initial contact area with a larger initial capacitance. At the same time, the highly twisted structures will help to partially release the deformations on the fiber during stretching strains. Consequently, the over‐twisted structure showed a reduced relative capacitance changes under strains. Considering the flexibility and sensing properties of the fibers, the strain sensors used in subsequent tests were constructed with 0.5 mm diameter fibers and a twist degree of 1256 rad m^−1^, unless otherwise specified.

**Figure 5:**
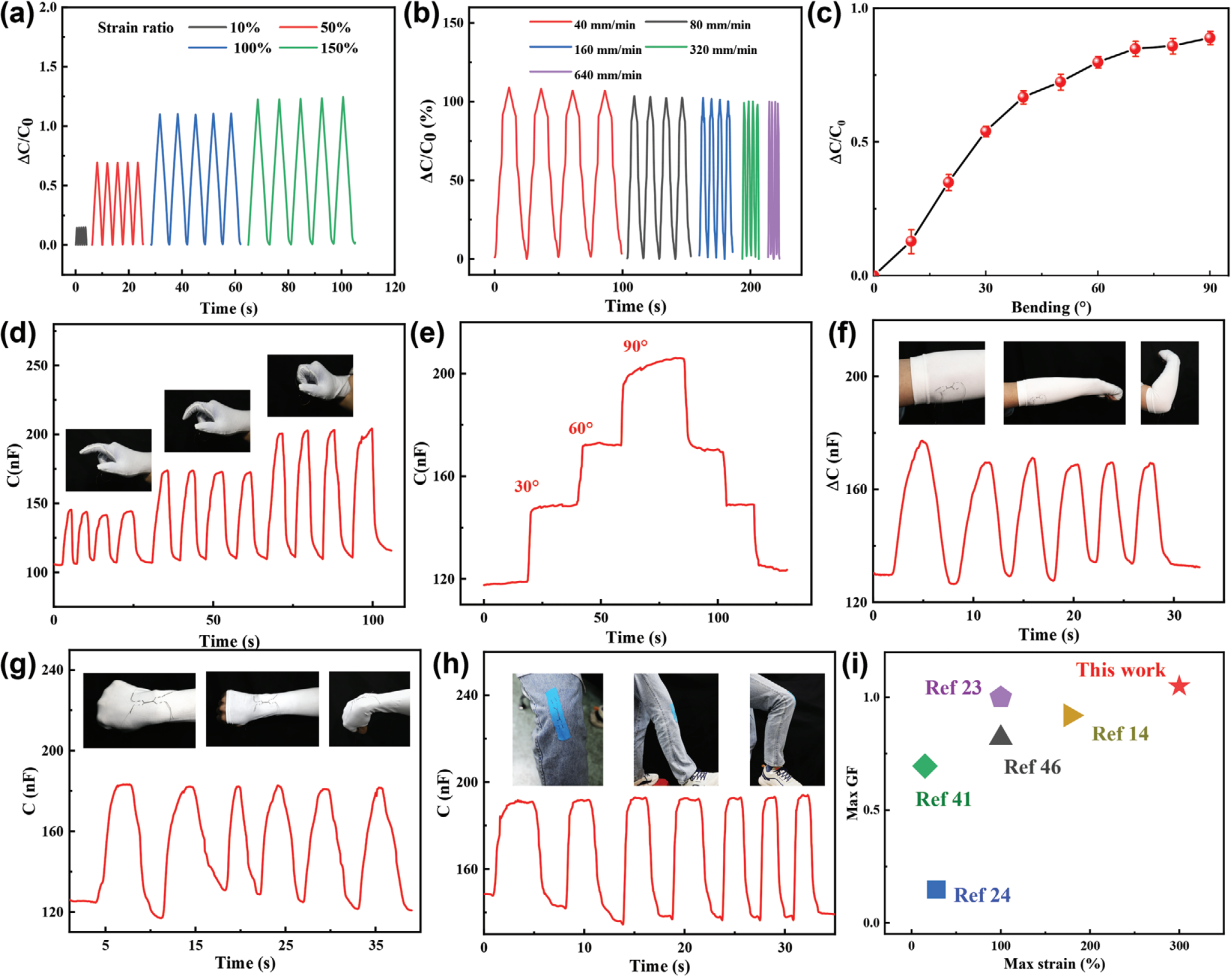
Dynamic performance and applications of the SICF‐based strain sensors: a) Change in capacitance of the strain sensor for a step strain of 10–150%. b) Change in capacitance of the strain sensor under a cyclic strain of 100% at various stretching speeds. c) Change in capacitance of the sensor as a function of finger bending angle. d) The capacitance response for 4 cycles of finger bending sensing at 30°, 60°, and 90°, respectively. e) The capacitance changes for continuous finger bending at different angles. f), g), and h) Strain sensors for wrist, elbow, and knee motion monitoring respectively. i) Comparison of the sensitivity and elasticity of the SICF‐based strain sensor with those capacitance sensors in previous reports.

The stability of the sensor's dynamic response is essential for wearable device applications like health monitoring. As illustrated in **Figure** **5a**, the sensor demonstrated excellent stability, repeatability, and capacity to differentiate between varying degrees of deformation across a range of 10% to 150% strain, making it suitable for monitoring different human motions. As illustrated in Figure 5b, the ΔC/C₀ peak remained nearly unchanged across varying stretching speeds, indicating the sensor's good stability. Furthermore, the SICF strain sensor demonstrated a rapid response to strain, with response and recovery times of ≈120 ms under rapid stretch, which is sufficient to meet the requirements of most human motion monitoring applications, as shown in Figure S8 (Supporting Information).

In the field of sports, dynamic posture monitoring of critical joints offers scientific data for correcting athletes' movements and preventing injuries. The strain sensors were sewn into a glove to sense finger movements, and the response curve of capacitance variation to finger bending angles is shown in Figure 5c. Due to the complex interaction of stretching and local compression on the sensor caused by finger bending, the response curve slightly differs from the pure stretching test in Figure 4c. Specifically, the capacitance experiences a more pronounced increase at smaller angles of finger bending. During the initial stage of bending, finger bending exerts both squeezing pressure and stretching on the fibers, resulting in closer contact between adjacent fibers at local locations. As the bending angle further increases, the squeezing force of the fingers on the fibers stabilizes, and the capacitance is primarily influenced by the elongation of the fibers. The response of the strain sensor to fixed bending angles of 30°, 60°, and 90° during cyclic bending and bending at varying angles are shown in Figure 5d,e, respectively. The strain sensor exhibits a high level of stability and accuracy in sensing finger‐bending motions. Using a similar mechanism, we sewed the sensors into wearable fabrics at the wrist, elbow, and knee, achieving motion detections of different human joints, as illustrated in Figure 5f–h, demonstrating the advantages of fiber‐based sensors for easy integration into wearable fabrics. In conclusion, the SICF‐based strain sensor exhibits enhanced sensitivity and a broader detection range compared to similar works,^[^ ^14^, ^23^, ^24^, ^26^, ^46^ ^]^ attributed to the high capacitive contribution of the EDL and the excellent stretchability of the fibers, as illustrated in Figure 5i and Table S1 (Supporting Information).

## 3 Conclusion

This study outlines a novel approach to developing ionic capacitive sensors based on 1D stretchable fiber materials. The fabricated stretchable fibers exhibit excellent electromechanical properties, ensuring reliable sensing performance of the sensors under complex deformation conditions. Leveraging the highly weavable nature of fibers, various sensor structures were devised to achieve sensing of stress and strain by direct integration with wearable fabrics. The designed sensors demonstrate robust stability and accuracy in response to stress and strain, enabling long‐term and continuous monitoring of human physiological signals and motion signals with comfortable wearable interfaces. We believe that more diverse sensor structures can be designed through the develop technology to suit various application scenarios, contributing to boosting the development of electronic textiles.

## 4 Experiment Section

### Fabrication of the I‐TPU Solution, Galinstan‐Ni Ink, and Core Fiber

TPU pellets (Desmopan; 1065AU), tetrahydrofuran (THF) (Shanghai Macklin Biochemical Co., Ltd.; AR, 99.0%), dimethylformamide (DMF) (Shanghai Macklin Biochemical Co., Ltd.; AR, 99.5%), 1‐Ethyl‐3‐Methylimidazolium Bis (trifluoromethylsulfonyl) Imide (Shanghai Macklin Biochemical Co., Ltd.; AR, 99.9%), Galinstan (Dongguan Huatai Metal Material Technology Co., Ltd.; 68.5% Ga, 21.5% In, 10% Sn) and nickel flakes (Nangong Jinnuo Welding Material Co., Ltd.; 5–10 µm; ≥99% trace metals basis) were purchased and used directly in the experiment without further processing.

TPU solutions were obtained by dissolving TPU pellets into THF and DMF. The weight ratio of TPU pellets, THF, and DMF was set at 1:2:1. The dissolving process was completed by a magnetic stirrer (JOANLAB HS5S) at 200 rpm for 12 h. Then, the ionic liquids were added to the TPU solutions with a weight ratio of 1:4. The solution was stirred at 300 rpm and 80 °C for 6 h with a magnetic stirrer to obtain homogeneous I‐TPU solutions. Galinstan‐Ni composite ink was prepared by ultrasonically mixing Galinstan and nickel flakes. For homogeneous mixing of 6.0 wt.% Ni in Galinstan, the mixture was continuously sonicated by a probe sonicator (LC‐JY16‐IID; Li‐Chen Technology Co., Ltd.)at 400 W for 15 min. The composite ink was then left overnight to stabilize. The core fibers are made by a thermal drawing process. TPU pellets were pressed at 110 °C for 15 min using a hot press (KY‐15YP small plate vulcanizing machine, supplied by Hefei United Technology Co., Ltd.) to obtain preforms with a diameter of 18 mm and a length of 140 mm. The preforms were thermal‐drawn into fibers in a triple‐temperature tube furnace at 110 °C by roller traction with a feed rate of 1 mm min^−1^ and an adjustable range of drawing speed from 18 to 180 mm min^−1^.

### Fabrication of Ionic Capacitive Fiber

Before coating, the core fibers were treated with a plasma cleaner (FARI GD‐5) at 200 W for 120 s to achieve a clean surface and Cu wires were twined at the end of fibers acting as connection electrodes. Then, the obtained core fibers were dipped into the Galinstan‐Ni inks and formed a uniform metallic film through a micro‐hole. The coated fibers were further dipped into the I‐TPU solutions to assemble the sensor fibers. Finally, the sensor fibers were dried in an oven for 24 h at 60 °C.

### Characterization

SEM images and EDS were characterized by a field emission SEM (FE‐SEM, JSM 7500F). All optical micrographs were obtained by a microscope (OLYMPUS BX53 M). The capacitances were acquired by LCR (Anbai‐AT3818x). The pressures were measured by a force gauge (SH‐III‐20N from Nscing). All mechanical properties of the samples were tested on a universal testing system (JHY 5000).

All experiments on wearable sensors involving humans were conducted following the relevant laws and institutional guidelines and under approval from the School of Mechanical Engineering, Sichuan University. All human subjects provided written and informed consent before participation in the experiments.

## Conflict of Interest

The authors declare no conflict of interest.

## Supporting information



Supporting Information

## Data Availability

The data that support the findings of this study are available from the corresponding author upon reasonable request.
