# Supplementary material for: Stretchable and High‐Performance Fibrous Sensors Based on Ionic Capacitive Sensing for Wearable Healthcare Monitoring
Source: Adv Sci (Weinh). 2024 Nov 11;12(1):2412859. doi: 10.1002/advs.202412859 (PMC11714226; doi:10.1002/advs.202412859)
Supplement: Supplementary file 1 — Supporting Information [file ADVS-12-2412859-s001.docx]

**Stretchable and High-performance Fibrous Sensors Based on Ionic Capacitive Sensing for Wearable Healthcare Monitoring**

*Jiawei Liu^1^, Yan Yang^1^, Guangchuan Chen, Hongbiao Sun, Xin Xie, Yanfeng Hou, Lishen Zhang, Jinhui Wang, and Jiangxin Wang**

School of Mechanical Engineering, Sichuan University, Chengdu 610065, China

^1^ These authors contributed equally to this manuscript

^*^ Corresponding Email: [wangjiangxin@scu.edu.cn](mailto:wangjiangxin@scu.edu.cn)


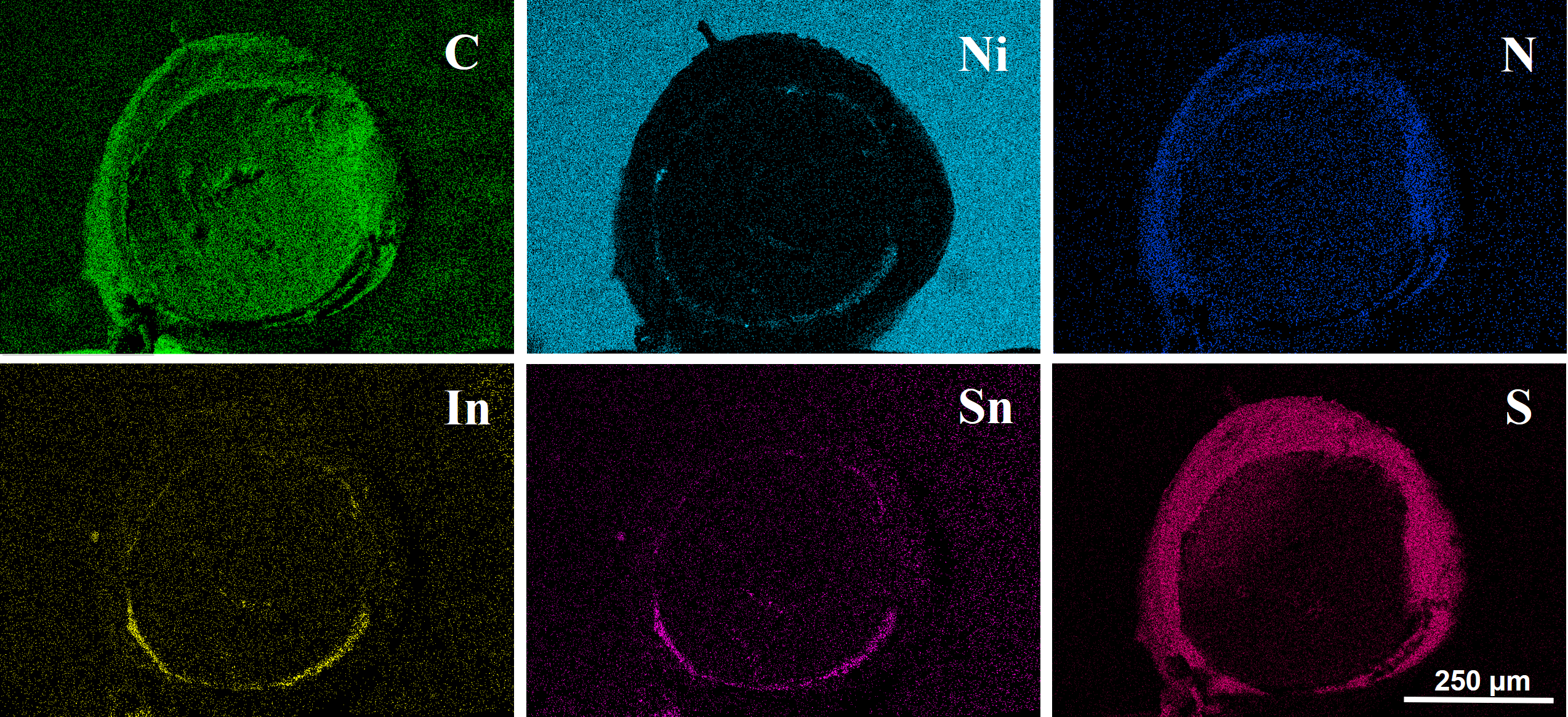


**Figure S1**. EDS images of SICF.


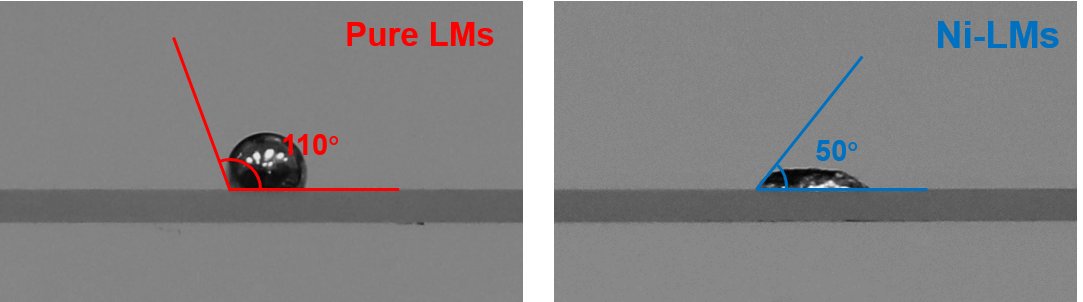


**Figure S2**. Contact angles of pure LMs and Ni-LMs on TPU substrates


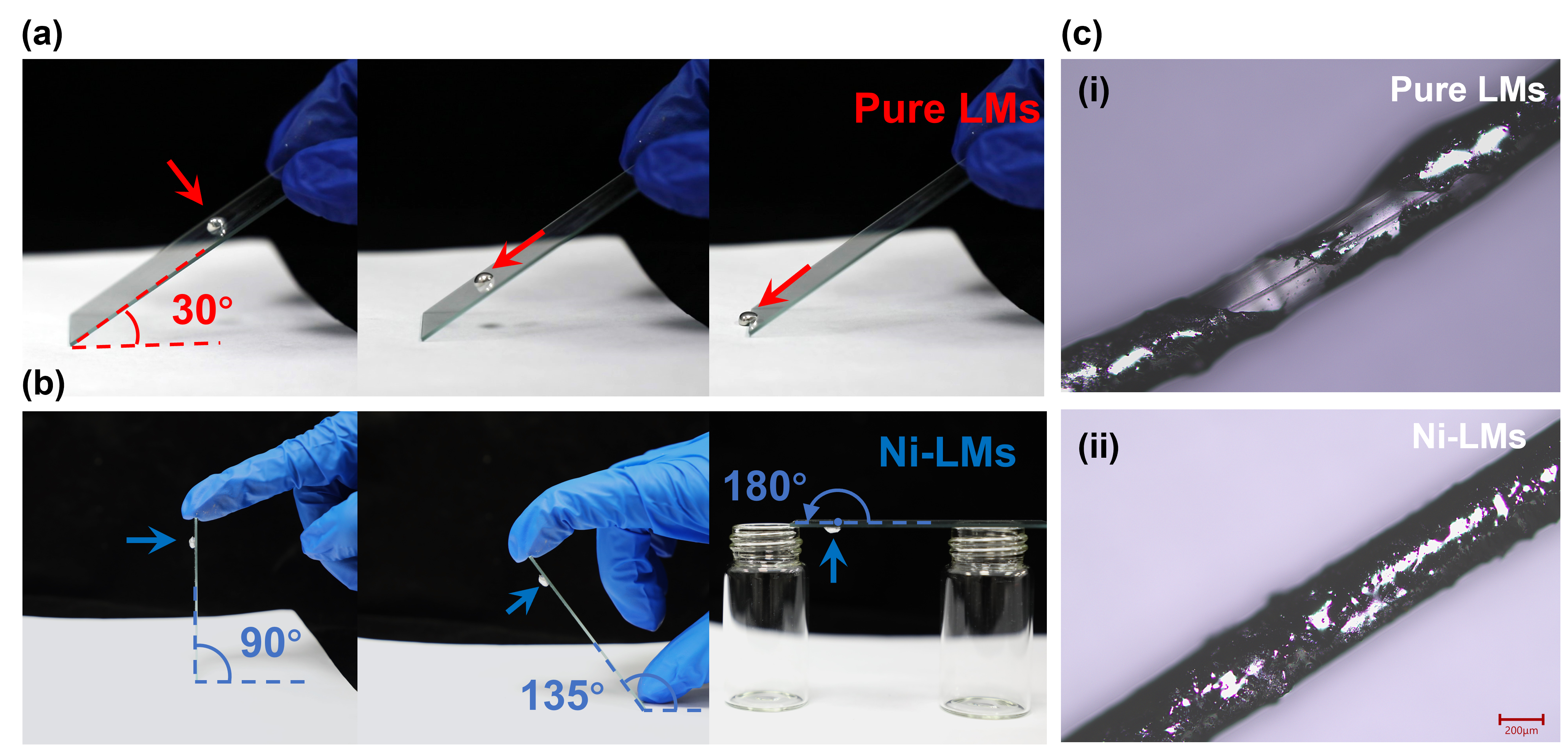


**Figure S3**. Sliding tests of pure LMs (a) and Ni-LMs (b) on TPU substrate; (c) Comparison of coating effect of pure LMs (i) and Ni-LMs (ii) on TPU fibers.

**Figure S4.** Stress-strain curve of the SICF.

By controlling the drawing and feeding rates, TPU fibers with diameters of 0.2 mm, 0.35 mm, and 0.65 mm were produced, which were subsequently fabricated into sensing fibers with the final diameters of 0.3 mm, 0.5 mm, and 0.8 mm, respectively. Sensors composed of smaller-diameter fibers exhibit enhanced sensitivity at lower pressures, as illustrated in **Figure S5**. However, the sensitivity declines rapidly as pressure increases. In contrast, sensors made from fibers with larger diameters demonstrate a higher detection limit and maintain good sensitivity across a broader pressure range.


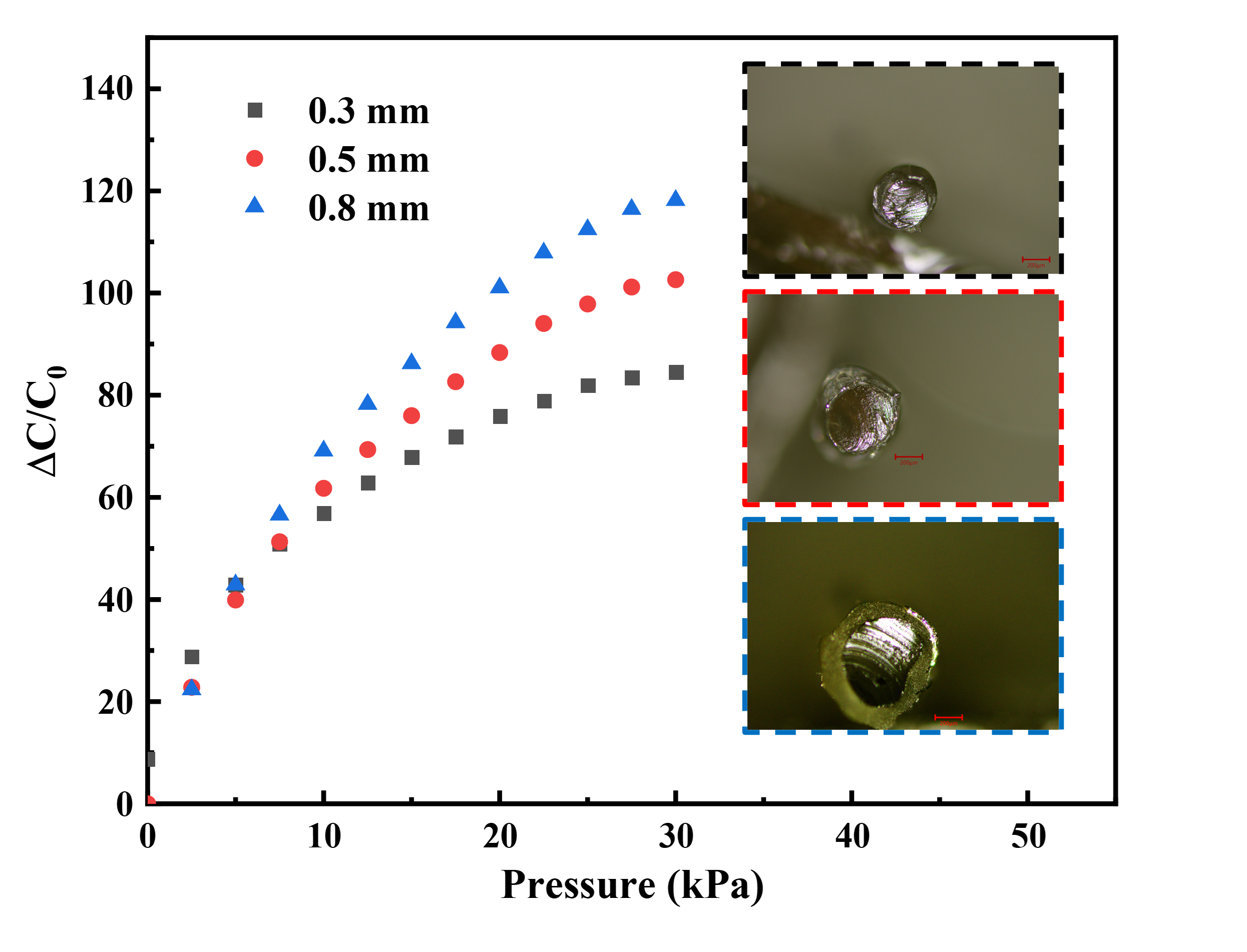


**Figure S5**. Pressure response of the pressure sensors consisting of 0.3mm, 0.5mm, and 0.8 mm diameter SCIFs.


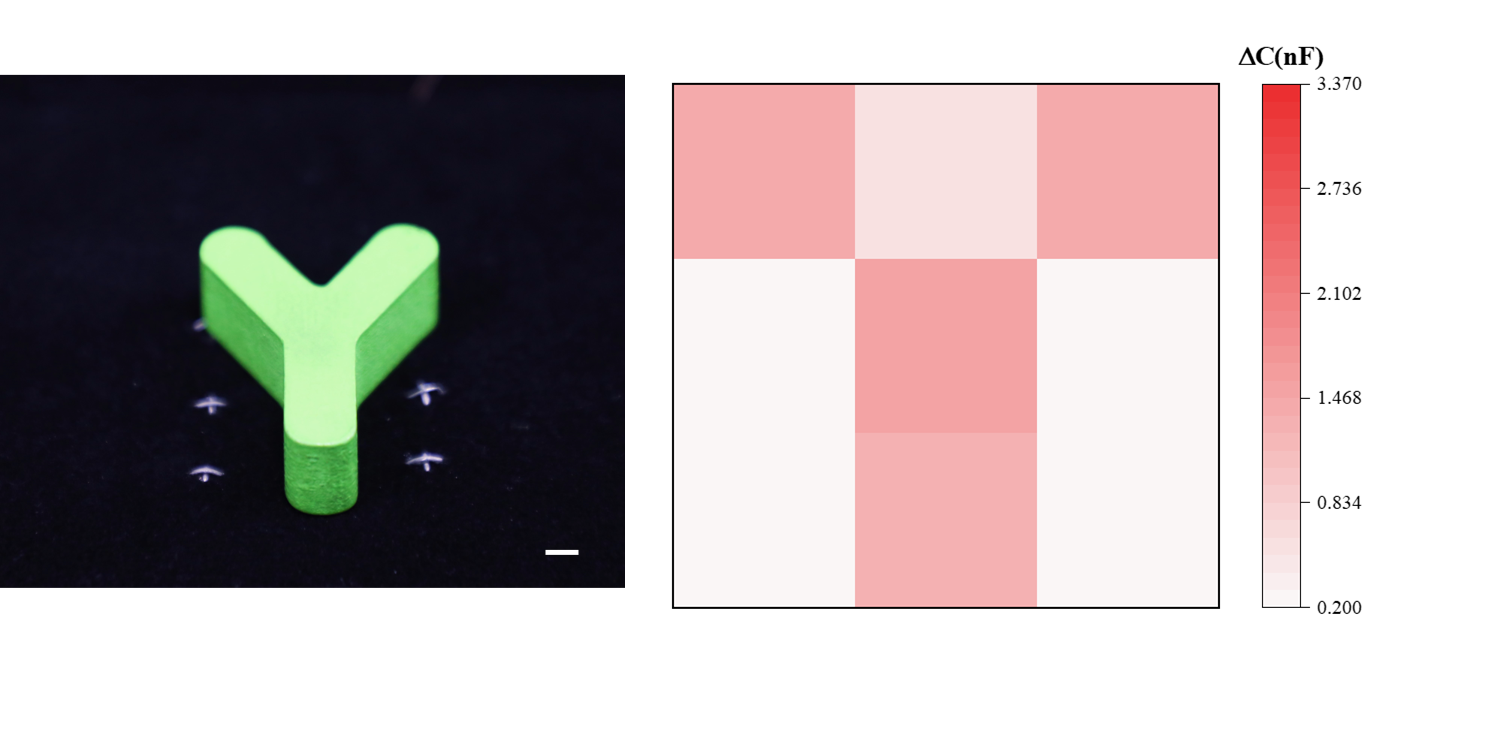


**Figure S6.** Photographs (i) and capacitance response (ii) of a 3*3 pressure sensing array for Y-shaped block sensing. Scale bar, 3 mm


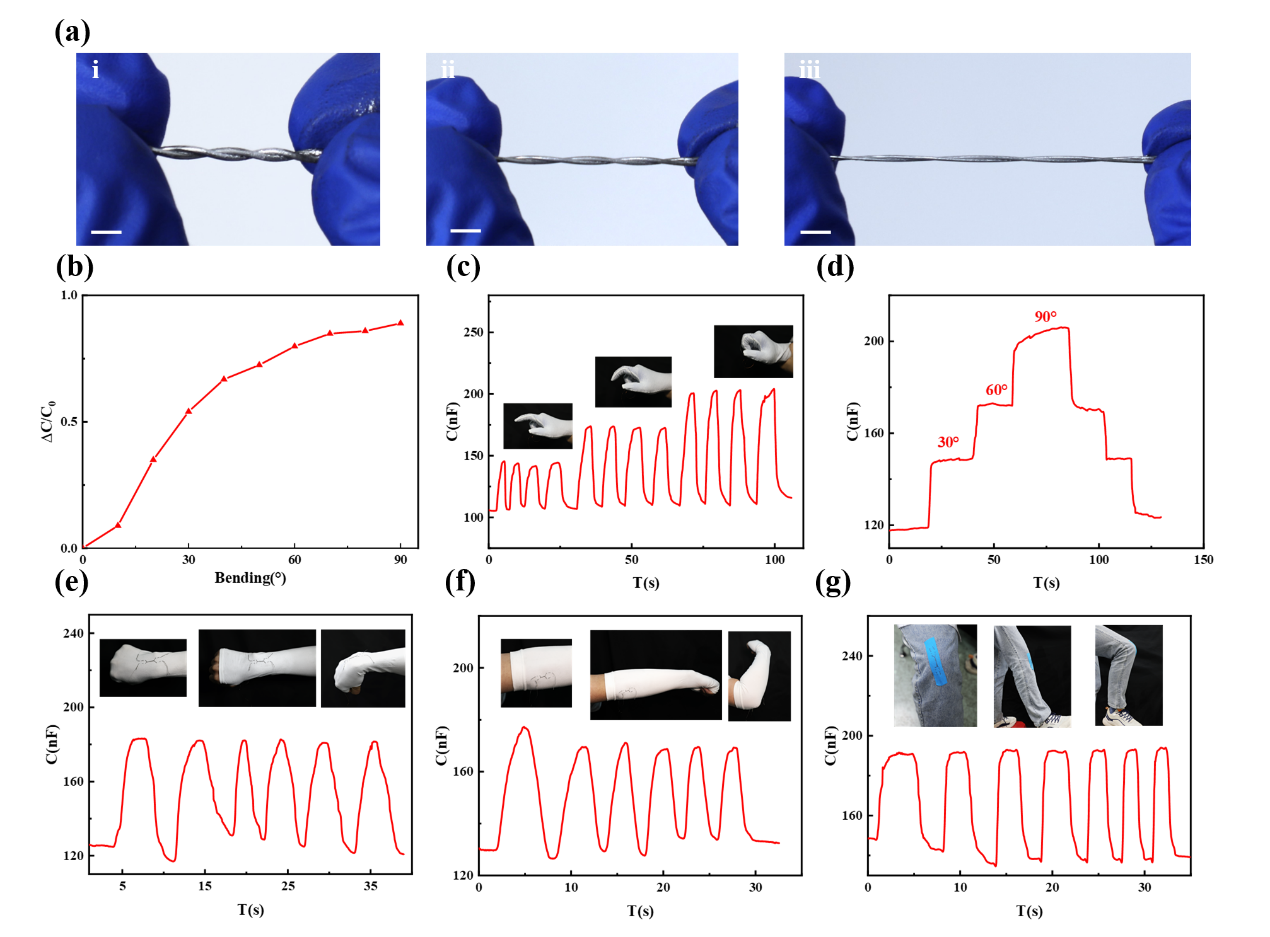


**Figure S7** Photographs of the strain sensor at its original length (i), approximately 50% stretching (ii), and approximately 100% stretching (iii) conditions. Scale bar, 2mm.

**Figure S8** The response and recovery times of the SICF strain sensor under rapid stretch.

**Table S1.** Compare with the reported capacitive fibrous strain and pressure sensors.

| Type of sensor | Material | Sensitivity | Detecting range | Response time | Ref. |
| --- | --- | --- | --- | --- | --- |
| 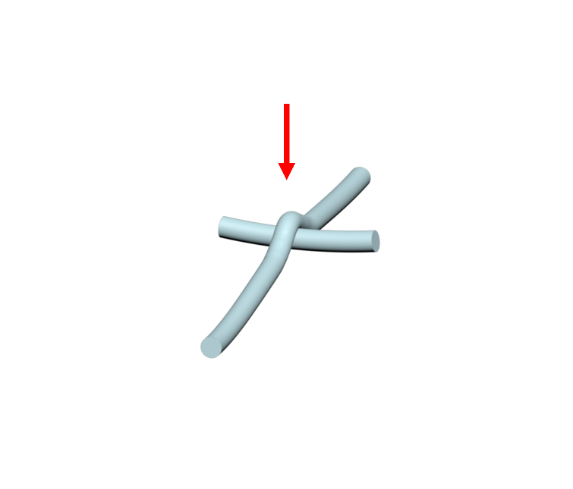  Stress sensor | Semi-solid metal/I-TPU | 7.21 kPa^-1^ below 6 kPa, 3.65 kPa^-1^ below 16 kPa, 1.54 kPa^-1^ below 30 kPa | 0 ~ 30 kPa | 60 ms / 30ms | This work |
|  | PU with high CNT content/PU with low CNT content | 1.28 kPa^-1^ below 2 kPa, 0.93 kPa^-1^ below 10 kPa, 0.76 kPa^-1^ below 25 kPa, 0.21 kPa^-1^ below 50 kPa | 0 ~ 50 kPa | 20 ms / 20 ms | 29 |
|  | AgNW-PEDOT/Ion-gel | 0.32 kPa^-1^ below 10 kPa, 0.07 kPa^-1^ below 50 kPa | 0 ~ 50 kPa | 227ms / 232 ms | 28 |
|  | Silver/cotton | 0.039711 kPa^-1^ below 0.85 kPa, 0.01922 kPa^-1^ below 35 kPa, 0.701 MPa^-1^ above 35 kPa | > 3.6 Pa | 110ms / 230 ms | 26 |
|  | AgNP-SBS/PDMS | 0.21 kPa ^-1^ below 2 kPa, 0.064 kPa^-1^ above 2 kPa | 0 ~ 10 kPa | >150 ms | 24 |
|  | CNT-PU/PU | 1.54 kPa^-1^ below 2 kPa, 0.47 kPa^-1^ below 10 kPa, 0.19 kPa^-1^ below 20 kPa | 0 ~ 20 kPa | 60 ms /60 ms | 30 |
|  | AgNW-BC/PDMS | 5.49 kPa^-1^ below 0.5 kPa, 0.65 kPa^-1^ below 7 kPa, 0.76 kPa^-1^ below 25 kPa, 0.01 kPa^-1^ below 460 kPa. | 0 ~ 460 kPa | 75 ms | 43 |
| 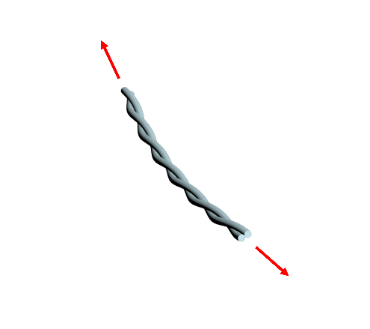  Strain sensor | Semi-solid metal/I-TPU | 0.23~1.05 | 0~300% |  | This work |
|  | LM/elastomeric polymer | 0.66 ~ 0.82 | 0~100 % |  | 46 |
|  | Ag/PDMS | <0.15 | 0~27.5% |  | 24 |
|  | Silver/cotton | 0.695 | 0~ 15% |  | 41 |
|  | LM/silicone | 1.07 | 0~100% |  | 23 |
|  | Ag/TPU/  BTO@Ecoflex | 0.924 | 0~178% |  | 14 |
